# Participation of RecJ in the base excision repair pathway of *Deinococcus radiodurans*

**DOI:** 10.1093/nar/gkaa714

**Published:** 2020-09-01

**Authors:** Kaiying Cheng, Ying Xu, Xuanyi Chen, Huizhi Lu, Yuan He, Liangyan Wang, Yuejin Hua

**Affiliations:** MOE Key Laboratory of Biosystems Homeostasis & Protection, Institute of Biophysics, College of Life Sciences, Zhejiang University, China; MOE Key Laboratory of Biosystems Homeostasis & Protection, Institute of Biophysics, College of Life Sciences, Zhejiang University, China; MOE Key Laboratory of Biosystems Homeostasis & Protection, Institute of Biophysics, College of Life Sciences, Zhejiang University, China; MOE Key Laboratory of Biosystems Homeostasis & Protection, Institute of Biophysics, College of Life Sciences, Zhejiang University, China; MOE Key Laboratory of Biosystems Homeostasis & Protection, Institute of Biophysics, College of Life Sciences, Zhejiang University, China; MOE Key Laboratory of Biosystems Homeostasis & Protection, Institute of Biophysics, College of Life Sciences, Zhejiang University, China; MOE Key Laboratory of Biosystems Homeostasis & Protection, Institute of Biophysics, College of Life Sciences, Zhejiang University, China

## Abstract

RecJ reportedly participates in the base excision repair (BER) pathway, but structural and functional data are scarce. Herein, the *Deinococcus radiodurans* RecJ (drRecJ) deletion strain exhibited extreme sensitivity to hydrogen peroxide and methyl-methanesulphonate, as well as a high spontaneous mutation rate and an accumulation of unrepaired abasic sites *in vivo*, indicating the involvement of drRecJ in the BER pathway. The binding affinity and nuclease activity preference of drRecJ toward DNA substrates containing a 5′-P-dSpacer group, a 5′-deoxyribose-phosphate (dRP) mimic, were established. A 1.9 Å structure of drRecJ in complex with 5′-P-dSpacer-modified single-stranded DNA (ssDNA) revealed a 5′-monophosphate binding pocket and occupancy of 5′-dRP in the drRecJ nuclease core. The mechanism for RecJ 5′-dRP catalysis was explored using structural and biochemical data, and the results implied that drRecJ is not a canonical 5′-dRP lyase. Furthermore, *in vitro* reconstitution assays indicated that drRecJ tends to participate in the long-patch BER pathway rather than the short-patch BER pathway.

## INTRODUCTION

The base excision repair (BER) pathway protects cells from the deleterious effects of endogenous and exogenous DNA damage induced by hydrolysis, reactive oxygen species, ionising radiation, and strong alkylating agents (1). The general BER process starts with a specific DNA glycosylase detecting and removing lesions (2,3). The resultant abasic (AP) site is recognised and incised by AP endonucleases at the 5′ side of the AP site to generate a 3′-hydroxyl (–OH) and 5′-deoxyribose-phosphate (–dRP), or by dRP lyases at the 3′ side of the AP site to generate a 3′-α,β-unsaturated aldehyde (–UA) and a 5′-monophosphate (–P) (4). In the short-patch BER pathway, further cleaning of the resultant 5′-dRP and 3′-α, β-unsaturated aldehyde is mediated by 5′-dRP lyases and AP endonucleases, respectively. A DNA polymerase fills the gap with a single nucleotide, and a DNA ligase seals the nick (5). By contrast, in the long-patch BER pathway, the DNA polymerase continues DNA synthesis from the 3′-hydroxyl for a few nucleotides with strand displacement, followed by degradation of the displaced strand by a flap endonuclease or 5′-3′ exonuclease (5).

*Deinococcus radiodurans* has strong resistance to agents that damage DNA, including radiation and oxidants (6). The BER pathway in *D. radiodurans* has not been well characterised. At the beginning of the BER pathway in *D. radiodurans*, alkylated bases are presumably removed by two AlkA glycosylases (DR2074 and DR2584), whereas deaminated and oxidised bases are removed by an ensemble of nine DNA glycosylases (7). *Deinococcus radiodurans* contains three homologs of endonuclease III (DR0928, DR2438 and DR0289), two of which possess both DNA glycosylase and AP lyase activities (8). However, none of them contribute to ionizing radiation and hydrogen peroxide resistance (9). In addition, *D. radiodurans* also has a formamidopyrimidine DNA glycosylase (Fpg, DR0439) and an AP endonuclease (ExoIII/Xth, DR0354) (10,11). Homologs of some conserved enzymes that remove the 3′-α,β-unsaturated aldehyde or 5′-dRP, including *Escherichia coli* exonuclease I, *E. coli* exonuclease IX, *E. coli* endonuclease IV, *Bacillus subtilis* ligase D and *B. subtilis* Ku (12–18), are missing in *D. radiodurans*. Polymerase X (PolX) of *D. radiodurans*, which is homologous to the eukaryotic DNA polymerase β (Pol β) (19,20), displays 5′-dRP lyase activity, and is believed to participate in the short-patch BER pathway (21). *Deinococcus radiodurans* also contains RecJ, a 5′−3′ single-stranded DNA (ssDNA)-specific exonuclease enzyme that is supposed to degrade abasic residues during base excision repair (22). RecJ is also believed to be involved in other DNA repair processes, including resecting DNA ends in the RecFOR homologous recombination pathway (23–25), reducing homology-facilitated illegitimate recombination events (26,27), rescuing stalled replication forks (28–31) and mediating the excision step during mismatch repair (32,33). The structure of *D. radiodurans* RecJ (drRecJ) in complex with its DNA substrate and its 5′-end resection mechanism have been explored (34). Herein, we determined the structure of drRecJ in complex with a DNA substrate containing a 5′-P-dSpacer group, a tetrahydrofuran derivative 5′-dRP mimic. A 5′-P binding pocket was identified, along with a possible 5′-dRP sensor motif. The mechanism for 5′-dRP catalysis was addressed based on structural and biochemical data. Biochemical and functional assays indicate that drRecJ is involved in the long-patch BER pathway rather than the short-patch BER pathway.

## MATERIALS AND METHODS

### Hydrogen peroxide (H_2_O_2_) survival rate

*Deinococcus radiodurans* wild-type (WT) strain R1 and its derivatives were grown at 30°C either in TGY broth (0.5% tryptone, 0.1% glucose, 0.3% yeast extract) or on TGY agar plates (TGY broth with 1.25% agar). Cells were grown to early exponential phase, equating to an optical density at 600 nm (OD_600_) of 0.6−0.8). After a series of dilutions, cultures were treated with different concentrations (ranging from 5 to 40 mM) of H_2_O_2_ for 30 min. Before being plated or dotted onto TGY plates, residual H_2_O_2_ was diluted with excess catalase. Plates were cultured for 2−3 days at 30°C, and colonies were counted. Cells without H_2_O_2_ treatment were used as controls. Three replicates were performed for each strain.

### Methyl-methanesulphonate (MMS) survival rate

MMS treatment was performed according to a previously reported method with some modifications (11). Cells were grown to early exponential phase (OD_600_ = 0.6−0.8). After treatment with different concentrations (0, 10 and 30 mM) of MMS for 15 min, cells were washed with autoclaved phosphate-buffered saline (PBS) buffer, serially diluted 10-fold at each step, and plated on TGY agar plates. Plates were cultured for 2−3 days at 30°C.

### Spontaneous rifampicin resistance rate

Assays were performed according to a previously reported method (35). Cells were grown to early exponential phase (OD_600_ = 0.6−0.8). After a series of dilutions, cultures were plated on TGY agar plates containing 50 μg/ml rifampicin (Sigma-Aldrich, St. Louis, MO, USA) or plated onto blank TGY agar plates as a control. After culturing for 2−3 days at 30°C, the number of colonies was counted, and the frequency of Rif^r^ mutations was determined by calculating the number of Rif^r^ colonies divided by the total number of viable cells on the blank plate. Three replicates were performed for each strain. Because the mutations that result in Rif^r^ are clustered within two small regions of the *rpoB* gene, they can be analysed using two primer pairs for amplifying and sequencing. Chromosomal DNA was isolated and amplified by Prifr-F and Prifr-R (36), and the resulting DNA fragments were collected for direct sequencing by the same primers. A summary of the sequencing results is shown in Table 1.

**Table 1.** Distribution of mutations leading to Rif^r^ in *D. radioduran* wild type and *△RecJ* strain

Amino acid change	Base pair change	Wild-type strain (50 colonies for sequencing)	*△RecJ* (50 colonies for sequencing)
Deletion (1310–18 bp)	-	14	2
Deletion (1318–26 bp)	-	9	0
S438P	AT→GC	8	9
L440P	AT→GC	2	8
L440R	AT→CG	2	4
D445N	GC→AT	5	3
H455Y	GC→AT	1	5
H455P	AT→CG	8	11
H455N	GC→TA	0	2
G463R	GC→AT	0	2
G499S	GC→AT	1	4
Total point mutations		27	48
Point mutations/total mutations		0.54	0.96

### AP site quantification assay

AP quantification assays were carried out using the protocol supplied with the Oxidative DNA Damage Quantitation Kit for AP site detection (Cell Biolabs, Inc., San Diego, CA, USA). Before measurements, cells were grown to early exponential phase (OD_600_ = 0.6−0.8) and treated with or without 40 mM H_2_O_2_ for 30 min. Cells were collected and washed twice. Genomic DNA was extracted from different samples using a TIANamp bacteria genomic DNA kit (TIAGEN Biotech, Beijing, China). The aldehyde reactive probe (ARP) that reacts specifically with an aldehyde group on the open-ring form of AP sites (ARP-derived DNA) was detected with a streptavidin-enzyme conjugate. The quantity of AP sites was determined using a BioTek plate reader (BioTek, Beijing, China) at 450 nm by comparing with a standard curve generated using predetermined AP sites. All DNA samples and standards were assayed using three duplicates.

### Protein purification and crystallisation

The procedures for drRecJ protein expression, purification, crystallisation and structure determination were performed as described previously (34). In brief, transformed *E. coli* Rosetta (DE3) cells were grown at 37°C in LB medium containing 50 mg/ml kanamycin to an OD_600_ of 0.6−0.8. Protein expression was induced for 5 h at 30°C by adding isopropyl-β-d-thiogalactopyranoside (IPTG) at a final concentration of 0.5 mM. After harvesting, cells were resuspended in lysis buffer (20 mM Tris–HCl pH 7.5, 1 M NaCl, 5% w/v glycerol, 3 mM β-mercapto ethanol and 10 mM imidazole), lysed by sonication, and centrifuged at 15 000 g for 30 min at 4°C. The supernatant was purified using a HisTrap HP column (GE Healthcare, Fairfield, CT, USA), equilibrated with buffer A (20 mM Tris–HCl pH 7.5, 1 M NaCl, 5% w/v glycerol and 10 mM imidazole), washed with 30 mM imidazole, and finally eluted with 300 mM imidazole. After tag removal using tobacco etch virus protease, the protein was loaded onto an MBP Trap HP column (GE Healthcare) to remove the uncleaved protein. The flow-through fractions were collected and loaded onto a Heparin HP column (GE Healthcare) pre-equilibrated with buffer B (20 mM Tris–HCl pH 7.5, 100 mM NaCl, 1 mM DTT and 5% w/v glycerol). Fractions containing drRecJ protein were eluted with a linear gradient from 100 to 500 mM NaCl. The protein was finally purified using a Superdex 200 10/300 GL column (GE Healthcare) with buffer C comprising 20 mM Tris–HCl pH 7.5, 100 mM NaCl, 0.1 mM ethylenediamine tetraacetic acid (EDTA) and 1 mM dithiothreitol (DTT), and stored at −80°C. The construction of drRecJ point mutants (R109A, H160A, R280A, K369A, S371A and R373A) has been reported previously (34). All drRecJ mutants were purified in the same way.

For complex crystallisation, the catalytically inactive drRecJ mutant (H160A) was mixed with DNA (5′-dRP-TTTTT) at a 1:2 molar ratio and concentrated to ∼18 mg/ml. Crystals were grown in 1.4 M Li_2_SO_4_, 100 mM MES (pH 6.5), 2.5 mM MnCl_2_ and 0.1 mg/ml SSB-Ct (EDDLPF) peptide. Cryocooling was achieved by stepwise soaking of crystals in reservoir solution containing 10%, 20% and 30% (w/v) glycerol for 3 min and flash freezing in liquid nitrogen. Diffraction data were recorded at the BL17U beamline of the Shanghai Synchrotron Radiation Facility (Shanghai, China), and were integrated and scaled using the XDS suite (37). Structures were determined by molecular replacement using drRecJ (PDB code: 5F55) as the search model (34). Structures were refined using PHENIX (38) and interspersed with manual model building using COOT (39).

For expression of drPolX, drXth, drPolA, drPolA(D119/120A), drPolA-C (drPolA 5′−3′ exonuclease domain truncated mutant), and drLigA, pET28a expression vectors were constructed and transformed into *E. coli* Rosetta (DE3) competent cells. All primers are listed in Supplementary Table S1. Protein expression was induced for 20 h at 16°C by adding IPTG at a final concentration of 0.2−0.4 mM. Briefly, drPolX was purified using a HisTrap HP column (GE Healthcare), a HiTrap Heparin column (GE Healthcare) and a Superdex 75 10/300 GL column (GE Healthcare). drXth and drLigA were purified using a HisTrap HP column (GE Healthcare), a HiTrap Q column (GE Healthcare), and a Superdex 75 10/300 GL column (GE Healthcare). drPolA, drPolA(D119/120A) and drPolA-C were purified using a HisTrap HP column (GE Healthcare), a HiTrap Heparin column (GE Healthcare), and a Superdex 200 10/300 GL column (GE Healthcare). All proteins were stored in 100 mM NaCl, 20 mM Tris–HCl (pH 7.5), 2 mM DTT, and 20% (w/v) glycerol at −80°C.

### Electrophoretic mobility shift assays

DNA binding assays were carried out according to a previously reported method (34), with some modifications. All DNA oligos with or without the 3′-end labeled by 6-carboxyfluorescein (6-FAM) were purchased from Sangon (Shanghai, China). The sequences of all oligos used in this work are listed in Supplementary Table S1. Regarding the synthetic 5′-dRP groups, 1′,2′-dideoxyribose (a tetrahydrofuran derivative in which a methylene group occupies the 1 position of 2′-deoxyribose) was used instead of 2′-dideoxyribose to introduce a stable AP site within the DNA substrate (i.e. a dSpacer modification). The 5′-P-dSpacer group was synthesised by linking a phosphate group to the 1′,2′-dideoxyribose group (dSpacer) at C5. Mass spectrometry (MS) data for the main oligos used in this study are included in Supplementary Figure S6. Samples containing 10 nM 3′-6-FAM-labeled ssDNA (KY08, KY10 and KY11) were incubated with different concentrations of RecJ (0, 1.25, 2.5, 5, 10, 20, 40 and 80 nM for WT; concentrations used for point mutants are listed in the figure legend) in a 10 μl reaction volume containing 50 mM TRIS-HCl pH 7.5, 150 mM KCl, 0.1 mg/ml bovine serum albumin (BSA), 1 mM DTT, and 5% (v/v) glycerol for 20 min at 30°C. Samples were separated on 8% native polyacrylamide gels in 1× Tris–borate–EDTA (TBE) buffer. Gels were imaged in fluorescence mode on a Typhoon FLA 9500 instrument (GE Healthcare). Bands were analysed using ImageJ software (National Institutes of Health, USA), and binding curves were created by GraphPad Prism 6 software (San Diego, USA). Nonlinear regression curves were fitted based on specific binding with a Hill slope. Dissociation constant (*K*_d_) values were calculated using the equation *Y* = *B*_max_ × *X^h^*/(*KD^h^* + *X^h^*).

### Nuclease activity assays

Nuclease activity assays were carried out according to a previously reported method (34) with some modifications. For a typical nuclease assay, 100 nM substrate was incubated with various concentrations (1.25−80 nM) of freshly prepared proteins in a 10 μl reaction with digestion buffer (50 mM Tris–HCl pH 7.5, 60 mM KCl, 0.1 mg/ml BSA, 1 mM DTT, 5% v/v glycerol and 0.1 mM MnCl_2_) for 20 min at 30°C. Reactions were stopped by incubating with 2× stop buffer (10 mM EDTA, 98% formamide, and 100 μg/ml Proteinase K) for 30 min at 100°C and flash-cooled. Reaction products were resolved on 15% polyacrylamide gels containing 7 M urea. Gels were imaged in fluorescence (FAM) mode on a Typhoon FLA 9500 instrument (GE Healthcare). Bands were analysed using ImageJ software (National Institutes of Health, USA). Substrates KY08, KY10, and KY11 (100 nM) were used to compare nuclease activity (0, 1.25, 2.5, 5, 10, 20, 40, and 80 nM for WT RecJ; concentrations used for point mutants are listed in the related figure legends) with different 5′-end modifications. Substrates KY04, KY05, KY18, KY19, KY20, KY21 and KY22 at 100 nM were used to test the required length of a 5′-ssDNA overhang for RecJ (160 nM) digestion initiation. KY28, annealed with KY29, KY30, KY31, KY32 or KY33, were used to test the required length of a 5′dRP-flap for RecJ (160 nM) digestion initiation. When drPolA-C was added, 1 mM dNTP and 5 mM MgCl_2_ were also added. Hairpin-structured or flap-structured DNA substrates were created in annealing buffer (10 mM HEPES pH 8.0, 50 mM NaCl, 0.1 mM EDTA) by heating at 95°C for 5 min and slowly cooling to 4°C. For steady-state measurements, 10 nM drRecJ was typically incubated with saturated substrate (KY08, KY10 or KY11 at 0−2 μM) in digestion buffer for 20 min at 30°C. All reactions were independently repeated at least three times. *K*_m_ and *k*_cat_ values were derived using the generalised nonlinear least squares method and the Michaelis-Menten equation, from which the apparent second-order rate constant (*k*_cat_/*K*_m_) was determined from a plot of the normalised initial rate (*v*/[E]) versus the substrate concentration ([S]). Kinetic parameters for drRecJ with different substrates are listed in Supplementary Table S2. For the 5′-^32^P-labeled substrates, 50 nM 20 nt poly (dA) substrates (with (KY24) or without a 5′-P-dSpacer group (KY23) were treated with 0, 1, 2, 4, 8 and 16 nM drRecJ in digestion buffer for 20 min at 30°C. Preparation of ^32^P-labeled DNAs is described below. PolX (50 nM) was used to create a 5′-P-dSpacer product, as a control. The drRecJ-treated products generated using the 5′-P-dSpacer substrate (KY26) were sent to Sangon (Shanghai, China) MS analysis.

### Preparation of ^32^P-labeled DNAs

Preparation of ^32^P-labeled DNA was carried out according to a previously reported method (40) with some modifications. A 20 μl sample of oligonucleotide (KY23 or KY24, 10 pmol/μl) was mixed with 2 μl γ-^32^P-ATP (10 mCi/ml; China Isotope & Radiation Corporation), 1.5 μl PNK enzyme (10 units/μl, New England Biolabs), 10 μl 10× PNK Buffer and H_2_O to a total volume of 100 μl. The liquid was mixed very gently and incubated for 60 min at 37°C, followed by heat-inactivation of the kinase by incubating at for 15 min 65°C. Next, 10 μl of 3 M NaOAc and 1 ml of 100% ethanol were added and incubated overnight at −20°C. The mixture was centrifuged at 12 000 g for 10 min, the pellet was washed with 1 ml 70% ethanol, centrifuged at 12 000 g, and air-dried for 30 min. DNA was resuspended in 100 μl H_2_O.

### Reconstitution of the long-patch BER pathway

A 541 bp DNA fragment (a random gene fragment from the *D. radiodurans* genome) was amplified by KY27 and KY27_PCR_R. KY27 labeled with 5′-6-FAM. To confirm purity, the DNA fragment was retrieved by agarose gel electrophoresis, then treated with 1 unit of *E. coli* UDG (New England Biolabs) for 30 min at 37°C in the presence of 100 mM NaCl, 50 mM Tris–HCl pH 7.5, 1 mM DTT, 5% glycerol and 0.1 mg/ml BSA. After incubating, the mixture was supplemented with 5 mM MgCl_2_, 1 mM dNTP, and 100 μM NAD+, and mixed with 20 nM drXth, 10 nM drRecJ, 50 nM drPolA (WT, D119/120A, or 5′−3′ exonuclease truncated domain drPolA-C), and 100 nM drLigA, when necessary. The final reaction system contained 10 nM substrate, 50 mM NaCl, 25 mM Tris–HCl (pH 7.5), 0.5 mM DTT, 2.5% glycerol, 0.05 mg/ml BSA, 5 mM MgCl_2_, 1 mM dNTP, 100 μM NAD+, and proteins. After further incubating for 30 min at 37°C, reactions were stopped by adding 2× stop buffer (10 mM EDTA and 98% formamide), boiling for 30 min, and flash-cooling. Reaction products were resolved on 8% polyacrylamide gels containing 8 M urea. Gels were imaged in fluorescence mode (FAM) on a Typhoon FLA 9500 instrument (GE Healthcare).

## RESULTS

### Deletion of RecJ reveals sensitivity to oxidising and alkylating agents, and an increase in the number of AP sites *in vivo*

**Figure 1.**
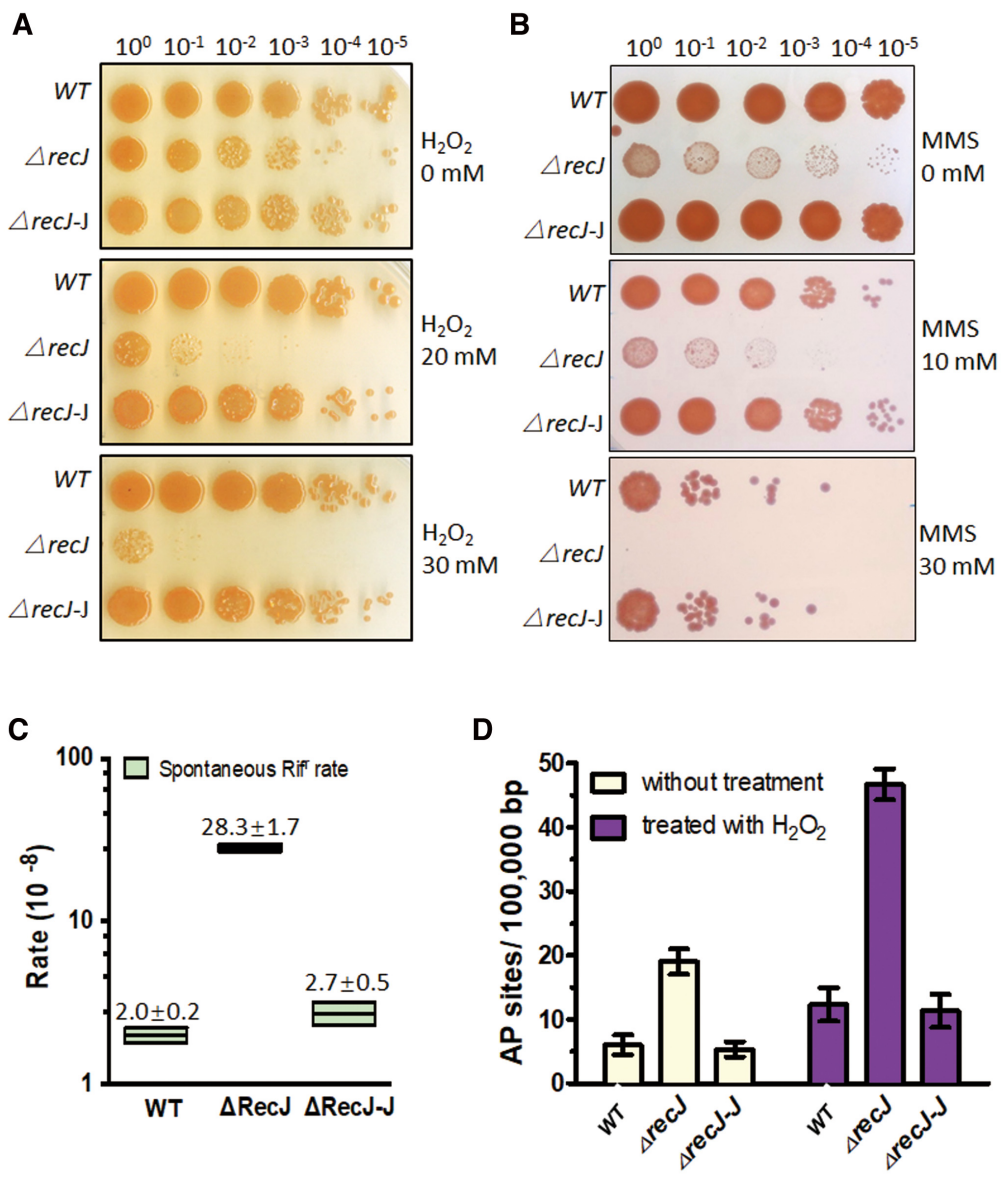
The phenotypes of drRecJ deletion mutant. (**A**) Wild type strain, drRecJ deletion mutant *△recJ* and *recJ* complemented strain *△recJ-J*, were treated with different concentrations of H_2_O_2_. Cells were diluted and dotted on plates. (**B**) Wild type strain, drRecJ deletion mutant *△recJ* and *recJ* complemented strain *△recJ-J*, were treated with different concentrations of MMS. Cells were diluted and dotted on plates. (**C**) The Spontaneous Rifampin resistance rates of wild type strain, *△recJ and* complemented strain *△recJ-J*. (**D**) The quantitation of DNA damage in the form of AP sites were measured in wild type strain, *△recJ* and *△recJ-J* under normal growing condition or with treatment of 20 mM H_2_O_2_.

H_2_O_2_ is an oxidising agent and MMS is an alkylating agent, and both can cause serious damage to DNA bases, resulting in AP sites after removal of oxidised or alkylated bases by DNA glycosylases. Therefore, H_2_O_2_ and MMS can, to some extent, be used as inducers of AP sites. Following exposure to different concentrations of H_2_O_2_ or MMS, the survival rate was compared between the *D. radiodurans* WT and *recJ* deletion (*ΔrecJ)* strains. As expected, the *ΔrecJ* strain was much more sensitive to H_2_O_2_ and MMS (Figure 1A and B). The survival fraction of *ΔrecJ* dropped ∼10^3^-fold and ∼10^4^-fold after treatment with 20 and 30 mM H_2_O_2_, respectively. The WT strain displayed robust resistance to H_2_O_2_ treatment, and survival was barely affected (Figure 1A and Supplementary Figure S1). On the other hand, the survival fraction of the *ΔrecJ* strain dropped ∼10^2^-fold and >10^5^-fold when treated with 10 and 30 mM MMS, respectively. By comparison, the survival fraction of the WT strain only dropped ∼10-fold after 10 mM MMS treatment and ∼10^3^-fold after 30 mM MMS treatment (Figure 1B). Complementation with RecJ could fully restore these survival defects caused by H_2_O_2_ or MMS treatment (Figure 1A, B, and Supplementary Figure S1).

Unrepaired AP sites can be bypassed by translesion synthesis, generating point mutations during semiconservative replication. The *rpoB*/Rif^r^ system, a canonical method for measuring the spontaneous mutation rate, was used to test whether drRecJ also influences the spontaneous mutation rate of *D. radiodurans*. The *recJ* deletion strain exhibited a ∼12-fold higher spontaneous rifampicin mutation rate than the WT strain (Figure 1C). Fifty Rif^r^ colonies of each strain were randomly picked and sequenced, and 27 of the 50 (54.0%) spontaneous mutations identified in the wt background were point mutations, while the rest were deletion mutations (Table 1). For *ΔrecJ*, 48 of the 50 (96.0%) spontaneous mutations were point mutations, many more than were identified in the WT strain.

To further confirm that RecJ participates in the BER pathway, we carried out assays to directly measure the number of AP sites *in vivo*. An enzyme-linked immunosorbent assay kit for the quantitation of DNA damage in the form of AP sites was used. This assay uses an ARP to specifically react with an aldehyde group on the open-ring moiety of an AP site, which allows the AP site to be labeled with biotin and detected by the streptavidin-enzyme conjugate. The ARP-DNA standard curve and raw data are provided in Supplementary Figure S2. Under normal growth conditions, *ΔrecJ* contained 4-fold more AP sites than the WT strain (Figure 1D). Treatment with H_2_O_2_ led to an increase in the number of AP sites in both *ΔrecJ* and WT strains, but again, *ΔrecJ* contained 4-fold more AP sites than the WT strain (Figure 1D). Therefore, RecJ was confirmed to participate in the removal of AP sites *in vivo*.

### drRecJ preferentially binds and digests DNA substrates containing 5′-dRP groups

**Figure 2.**
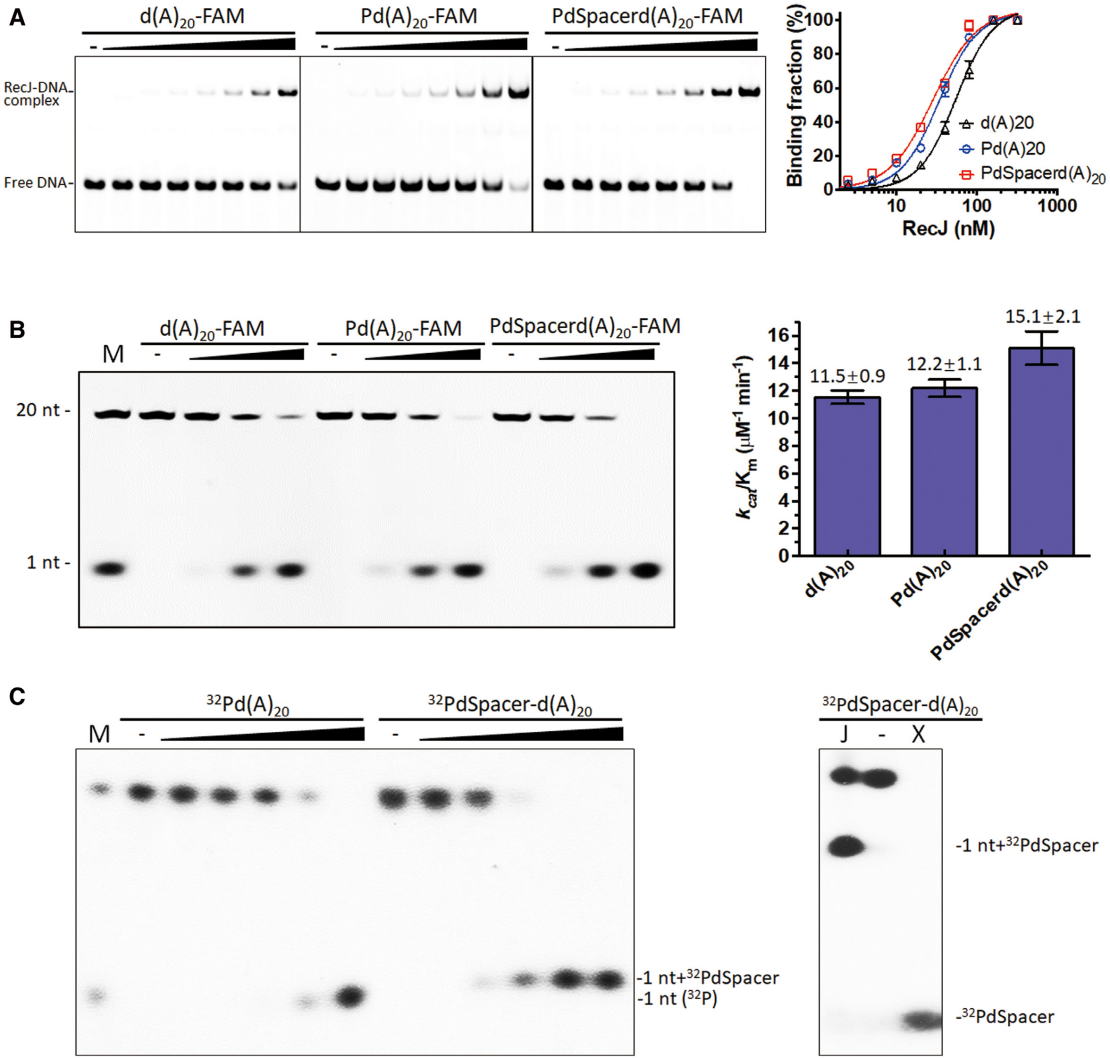
RecJ binding affinity and nuclease activity on ssDNA with different 5′ end modifications. (**A**) Different substrates with various 5′ modifications (10 nM), as shown at the top of the panel, were incubated with drRecJ (0, 1.25, 2.5, 5, 10, 20, 40 and 80 nM). The formed complexes were separated by native gel. Nonlinear regression curves were fitted based on Specific binding with Hill slope. (**B**) Different substrates with various 5′ modifications (100 nM), as shown at the top of the panel, were incubated with drRecJ (0, 5, 10 and 20 nM) in the presence of 100 nM Mn^2+^ and then separated by denaturing PAGE gel, the *k*_cat_*/K*_m_ was determined according to three replicates (see Materials and Methods) and shown as bar chart on the right. (**C)** Left: 50 nM 5′ ^32^P-labeled 20 nt poly (dA) substrates or 5′ ^32^P-dSpacer-labeled 20 nt poly (dA) were digested by 0, 1, 2, 4, 8 and 16 nM RecJ and analyzed on 20% denaturing PAGE gel; Right: 50 nM substrate containing 5′-^32^P-dSpacer group was treated with 16 nM RecJ (lane J) and 50 nM PolX (lane X).

It was reported that *E. coli* RecJ can release the 5′-terminal deoxyribose phosphate residues from incised AP sites in DNA (22). Here, a 20 nt poly (dA) with or without a 5′-end modification (containing a 5′-monophosphate group or 5′-P-dSpacer group, a tetrahydrofuran derivative 5′-dRP mimic) was used as a substrate for drRecJ to test its binding affinity and digestion efficiency (Figure 2A, B and Supplementary Table S2). The phosphorylation status of the 5′-end moderately enhanced the binding affinity and catalytic efficiency (*k*_cat_*/K*_m_); Kd values for 20 nt poly (dA), and 5′-P-poly (dA) were 54.9 ± 5.18 and 33.85 ± 2.83 nM, respectively; *k*_cat_*/K*_m_ values for 20 nt poly (dA) and 5′-P-poly (dA) were 11.5 ± 0.9 and 12.2 ± 1.1 μM^−1^ min^−1^, respectively. The 5′-P-dSpacer modification resulted in a stronger enhancement of both the binding affinity (*K*_d_ = 28.43 ± 3.1 nM) and catalytic efficiency (*k*_cat_*/K*_m_= 15.1 ± 2.1 μM^−1^ min^−1^) for RecJ, indicating that the substrate containing 5′-dRP was the optimum substrate for drRecJ. It is worth noting that *k*_cat_ values for the three substrates were almost the same (Supplementary Table S2), indicating similar catalytic turnover rates. Therefore, their catalytic efficiencies are mainly influenced by the *K*_m_ value, which reflects the enzyme-substrate affinity.

To confirm the exact products of drRecJ, 5′-^32^P-labeled or 5′-^32^P-dSpacer-labeled 20 nt poly (dA) was used for digestion analysis. The 5′-^32^P-dSpacer groups, which were created after treating with drPolX, served as controls. Product bands generated by drRecJ ran much slower than the 5′-^32^P-dSpacer band and slightly slower than the 1 nt band on the denaturing polyacrylamide gel, as expected for 1 nt plus 5′-^32^P-dSpacer (Figure 2C). Further MS analysis was carried out to confirm that the products generated by drRecJ were mainly dAMP and 5′-P-dSpacer-dAMP (Supplementary Figure S3), and the signal for the 5′-P-dSpacer group was minimal. These results implied that the catalytic mechanism of RecJ acting on substrates containing dRP groups is distinct from that of canonical dRP lyases; RecJ breaks the second phosphodiester bond and leaves a 5′dRP plus a nucleotide, while canonical dRP lyases break the first phosphodiester bond and leave only a dRP group.

### The 5′-dRP binding pocket and the RecJ catalytic mechanism

**Figure 3.**
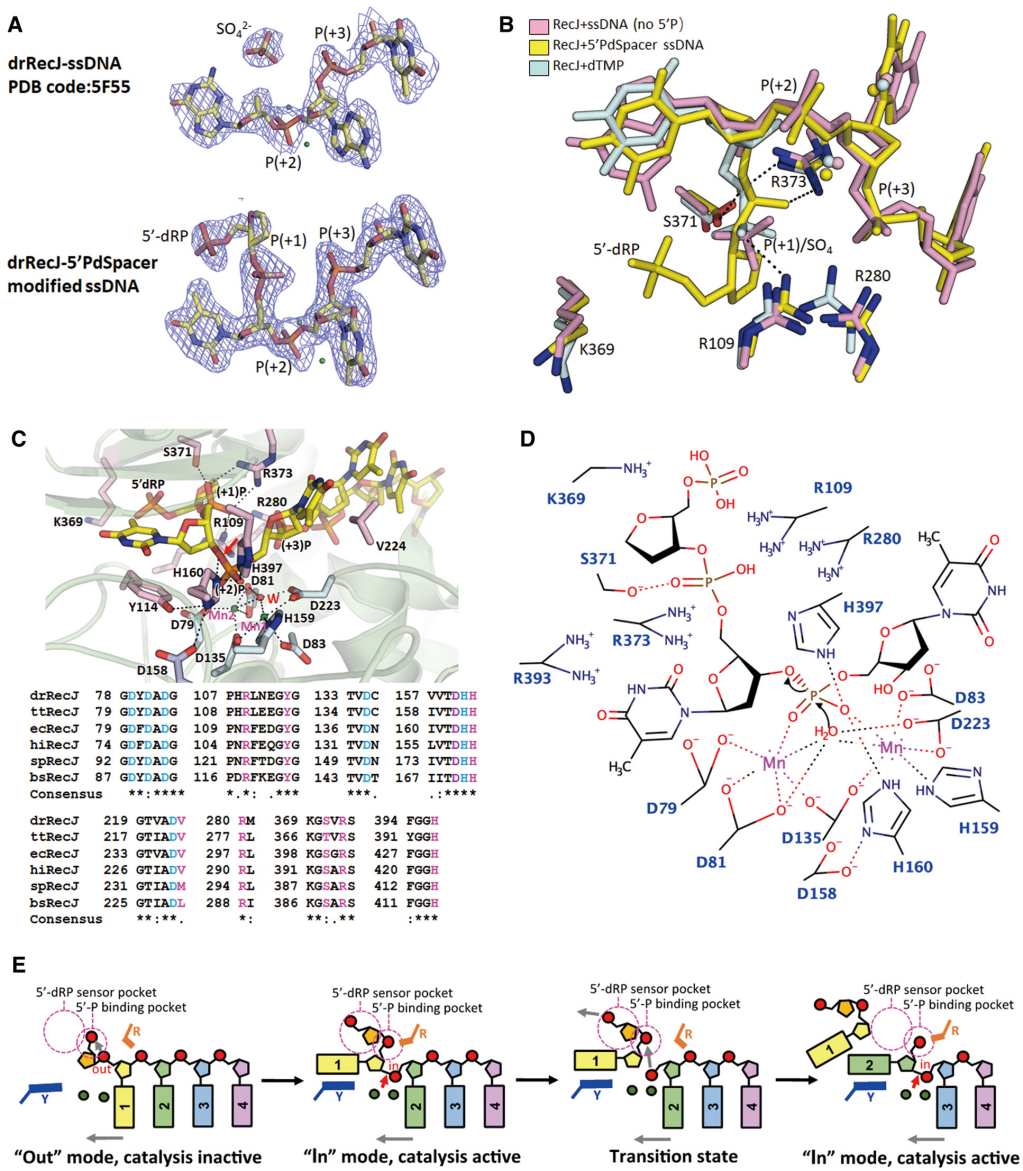
The 5′-P binding pocket and 5′-dRP sensor motif. (**A**) The density of ssDNA (PDB code: 5F55) or 5′-P-dSpacer modified ssDNA in the complex are compared. Substrates are shown as sticks; the Mn^2+^ ions are denoted by smudge nb_spheres. The *F*_o_*–F*_c_ map is contoured at 1.6σ level. (**B**) The superimposition of nucleotide binding sites of complex containing 5′-dRP modified ssDNA, ssDNA (PDB code: 5F55) and dTMP (PDB code: 5F54) . Nucleotides and side chains of amino acids are shown as sticks; The Mn^2+^ ions are denoted by nb_spheres. For complex with 5′ dRP modified ssDNA, both the substrates and amino acids are coloured yellow. For complex with ssDNA, both the substrates and amino acids are coloured pale cyan. For complex with dTMP, both the nucleotide and amino acids are coloured light pink. All the hydrogen bonds and metal-ion coordination are shown as black dashed lines. (**C**) The conserved residues around catalysis core of drRecJ. Upper, substrates and side chains of main amino acids are shown as sticks and other chains are shown as cartoon with 80% transparency. The residues participate in metal-ion coordination are coloured light pink and the residues participate in nucleotides interaction are coloured light blue. The Mn^2+^ ions are denoted by nb_spheres and coloured smudge, and the catalytic water molecule are shown as nb_sphere and coloured red. Bases of substrates are numbered. All the hydrogen bonds and metal-ion coordination are shown as black dashed lines. The red arrowhead indicates the position of P–O bond breakage. H160 was artificially created here in order to show the catalysis mechanism, which was mutated into alanine in real structure data. The hydrogen bonds are labeled as dashed lines. Below, sequence alignment shows conserved motifs/residues of RecJ catalysis core. dr, *Deinococcus radiodurans*; tt, *Thermus thermophilus*; ec, *Escherichia Coli*; *hi, Haemophilus influenza*; *sp, Streptococcus pneumoniae*; *bs, Bacillus subtilis*. Letters coloured blue represent metal binding/catalysis related residues. Letters coloured pink represent substrate translocation related residues. (**D**) Diagram showing the coordination of DNA substrate containing 5′-dRP and Mn^2+^ in the active site of drRecJ and the proposed catalytic mechanism. Mn1 and Mn2 are proposed to be responsible for the activation of the catalytic water molecule, followed by the production of the nucleophilic hydroxyl. The black arrowheads show the directions of nucleophilic attack. All the hydrogen bonds and metal-ion coordination are shown as dashed lines. (**E**) A model for catalysis and translocation of 5′-dRP DNA substrate in drRecJ nuclease core. The 5′-P binding pocket and 5′-dRP sensor motif are marked as pink dotted circles. The tyrosine (Y114) forms stack interaction with bases is labeled as Y and coloured blue. The three arginine residues (R109, R280 and R373) important for translocating phosphate group to the 5′-P binding pocket are labeled as R and coloured orange. Metals are shown as green circles. The red arrowhead indicates the position of P-O bond breakage. The grey arrowhead indicates the direction of substrate translocation.

To characterise how RecJ digests a DNA substrate containing an abasic residue, 5′-P-dSpacer-modified ssDNA was co-crystallised with the catalytically inactive RecJ protein (the H160A mutant). The crystal diffracted to a 1.9 Å resolution, and the structure was determined by molecular replacement using a previously solved drRecJ-ssDNA structure (PDB ID: 5F55) (34) as the initial model. Statistics for data collection and refinement are listed in Table 2. Some of the 5′-P-dSpacer groups might be flexible and/or be degraded during the crystallisation process, resulting in weak electron density in that area of the map. However, a clear (+1) phosphate group density was observed in the nuclease core (Figure 3A). Unlike the broken density in our previous structure (PDB ID: 5F55), which consisted of a separate sulphate radical and a substrate without 5′-P, the density connecting the first phosphate group and the first deoxyribose was continuous in this structure (Figure 3A). The (+1) phosphate group in this structure superimposes well with the monophosphate group of dTMP in the drRecJ-dTMP structure (PDB ID: 5F54) and the sulphate radical in the drRecJ-ssDNA structure (PDB ID: 5F55; Figure 3B) (34). Four conserved residues, R109, R280, S371 and R373, form hydrogen bonds with the (+1) phosphate group, resulting in a 5′-monophosphate binding pocket. Based on the position of the (+1) phosphate group and the weak density for 5′-P-dSpacer, a 5′-dRP group was built at the 5′-end of the DNA substrate. No obvious steric clash between the protein and nucleotide was observed, indicating that this area is suitable for 5′-dRP occupation (Figure 3B and C). Furthermore, a nearby conserved lysine (K369) might bind the monophosphate group of 5′-dRP during the product releasing process (Figure 3B and C). Alanine substitutions of R280 or S371 eliminated both the binding and catalysis preference for 5′-P-dSpacer-modified DNA or 5′-P-modified DNA (Supplementary Figures S4 and S5). Moreover, R109A, R373A, and K369A mutants suffered up to a two-fold decrease in catalytic activity for 5′-P-dSpacer-modified DNA, compared with substrates without this modification (Supplementary Figure S5). The R109A mutant displayed two-fold reduction in binding to 5′-P-dSpacer-modified DNA, while preferential binding was completely lost for R373A and K369A mutants (Supplementary Figure S4).

**Table 2.** Statistics from crystallographic analysis

	RecJ_d_-5′-PdSpacer-d(T)_5_
**Data collection**	
Space group	*P* 3_2_21
Cell dimensions *a*, *b*, *c* (Å)	106.140, 106.140, 164.560
Wavelength (Å)	0.9792
Resolution (Å)	30–1.90 (1.95–1.90)
*R*-merge (%)	7.1 (59.9)
*I*/*σI*	19.0 (1.9)
Completeness (%)	98.3 (99.8)
Redundancy	8.5
**Refinement**	
Resolution (Å)	30–1.90 (1.95–1.90)
No. reflections	83333
*R*_work_/*R*_free_ (%)	20.9 / 23.3
No. atoms (total)	5901
Protein/DNA	5281/111
Ligand/Ion	57
Waters	452
Bond length (Å)	0.008
Bond angle (°)	1.132
Ramachandran statistics favored (%)/outliers (%)	98.99/0

Values in parentheses refer to the highest resolution shell.

*R* factor = Σ||*F*(obs) – *F*(calc)||/Σ|*F*(obs)|.

*R*_free_ = *R* factor calculated using 5.0% of the reflection data randomly chosen and omitted from the start of refinement.

RecJ_d_ denotes catalytic inactive drRecJ (H160A).

Based on this newly solved structure, together with previously reported structures (PDB ID: 5F54 and 5F55) and the biochemical data described above, a simplified catalytic mechanism for phosphodiester bond hydrolysis by drRecJ has been proposed (Figure 3D and E). The (+1) phosphate group is recognised and binds the 5′-P binding pocket. A conserved tyrosine residue (Y114) holds the first base by forming a π-stacking interaction with it, which induces a ‘U-turn’ conformational change of the DNA backbone, and this converts the (+2) phosphate into the ‘in’ mode, placing it in the correct position for in-line attack. The significance of Y114 has been demonstrated by alanine displacement, which resulted in extremely reduced exonuclease processivity (34). Two Mn^2+^ ions are coordinated by five aspartate residues, one histidine, and the (+2) phosphate group. Alanine substitutions of these metal ion binding residues almost blocked the nuclease activity of drRecJ (34). A water molecule, located on the 5′ side of the phosphate group, bound by two Mn^2+^ ions, may be deprotonated by one of the metal ions, and subsequently acts as a nucleophile to attack the 3′-phosphate-ester bond. The completely inactive H160A mutant, which we used to grow complex crystals with different substrates (34), does not bind metal ions but might form hydrogen bonds with the (+2) phosphate group and assist in nucleophilic attack. D158 controls the position of the active site main chain backbone, and thereby not only directly affects the side chain orientation of D159, but also stabilises H160 in the correct position by forming hydrogen bonds with it. This explains why D158 plays an essential catalytic role despite being located far away from the metal ions, the water nucleophile, and the substrate. After hydrolysis, His397 may serve as a general acid to protonate the 3′-O leaving group and generate a 3′-hydroxyl group on the dT1 nucleotide. K369, located near the exit of the nuclease core, may capture the (+1) phosphate group and assist the release of dT1. The (+2) phosphate group is translocated by a bundle of basic residues (R109, R280 and R373) to the 5′-P binding pocket. dT2 then moves one step forward, the (+3) phosphate group is converted to the ‘in’ mode, and becomes ready for the next round of hydrolysis. Compared with the catalytic pattern of WT drRecJ, for which only single nucleotide products can be detected (Figure 2B), R109A, R280A and R373A mutants all yielded a number of bands on the gel (Supplementary Figure S5), indicating impaired nuclease processivity.

Regarding the 5′dRP-containing substrate, because there is no base linked to the 5′dRP group, Y114 can no longer induce the ‘U-turn’ conformational change of the DNA backbone. Therefore, the (+1) phosphate group is in the ‘out’ mode, which is catalytically incompetent, hence substrates can easily escape digestion and occupy the 5′-P binding pocket (Figure 3E). Such a mechanism can explain why only a small amount of the 5′-dRP group was detected after incubation with drRecJ (Figure 2C and Supplementary Figure S3).

### Reconstitution of the BER pathway *in vitro*

drRecJ alone could digest double-stranded DNA (dsDNA) with a 5′-end overhang no less than 4 nt or 3 nt plus a 5′-dRP group (Supplementary Figures S7 and S8), which is in agreement with the structural data showing that the helical gate of the ssDNA binding channel and the embedded catalytic core are separated by a distance equal to four bases (34). These results indicate that RecJ alone could not access the 5′-dRP end that is embedded in the duplex substrate. Meanwhile, drRecJ exhibited highly processive nuclease activity once initiated, and it could digest short DNA substrates to 1 nt products (Supplementary Figures S7 and S8), further implying that drRecJ does not participate to a large extent in short-patch BER pathway, which only removes one dRP group. On the other hand, the presence of *D. radiodurans* DNA polymerase I (drPolA) may assist drRecJ digestion initiation by generating a 5′-flapped ssDNA during the synthesis of a new strand, which is a key step in the long-patch BER pathway (Supplementary Figure S8).

**Figure 4.**
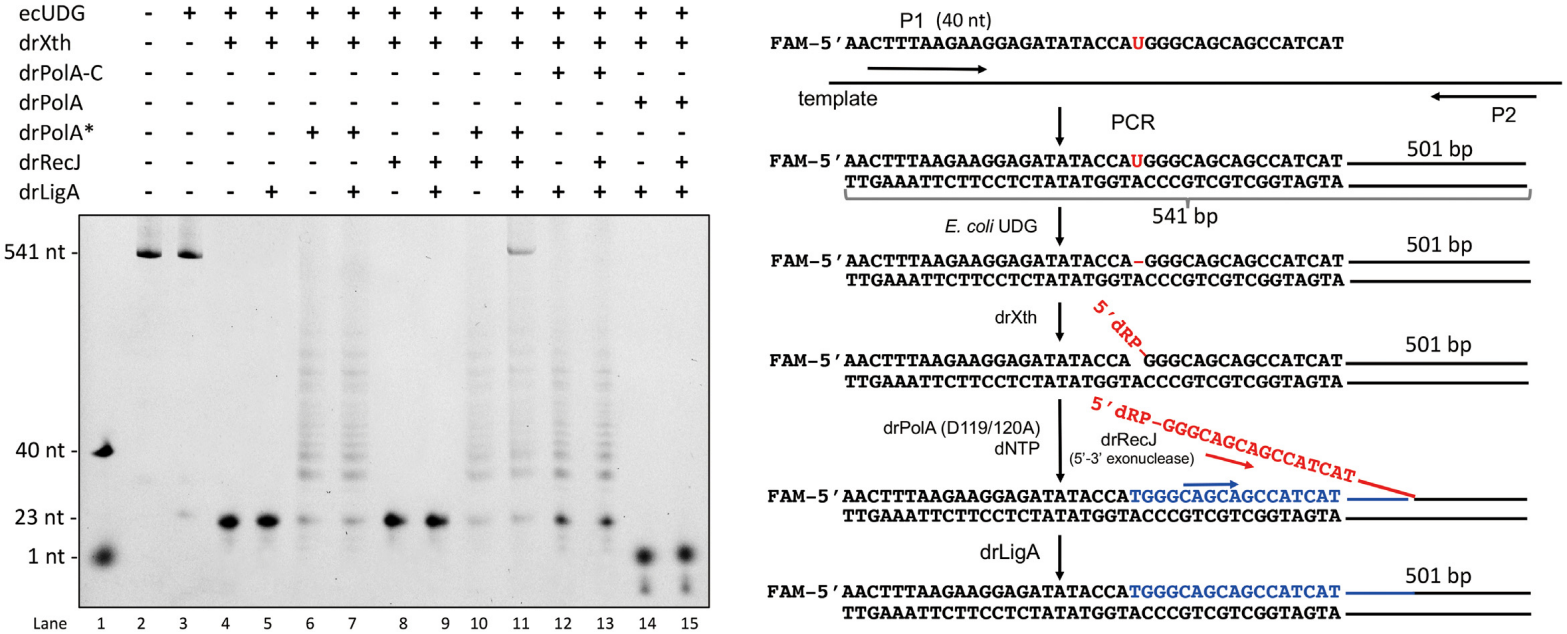
The reconstitution assay of the BER pathway *in vitro*. Left, the denature gel showing the reconstitution assay of the long-patch BER pathway. In the presence or absence of drXth, drRecJ, drPolA (or drPolA (D119/120A), or drPolA-C) and drLigA, DNA digestion, synthesis and ligation process were detected. Right, the diagram of the reconstitution assay (corresponding to the left gel, lane 11). 5′-6-FAM labeled uracil-containing DNA was used as upstream primer, together with an unlabeled downstream primer, to amplify a 541 bp DNA fragment. Fragments were treated with *E. coli* UDG, leaving an intact AP site. The detailed experiment was carried out as described in Materials and Methods. The resulting DNA was incubated with drXth, which incises 3′ end of the AP site, and create a 3′-OH and 5′-dRP group. Then, drPolA mediates DNA synthesis from 3′-OH and promotes a displacement of the downstream DNA strand, creates a 5′ flap. When the 5′ flap is long enough, drRecJ is supposed to recognize the 5′-dRP, digest the 5′ flap efficiently into DNA duplex region for a few hundred nucleotides, and leave a nick containing 5′-P. Such nick is ligated by drLigA.

To reconstitute the BER pathway *in vitro*, a 541 bp DNA fragment was amplified by a 5′-6-FAM-labeled uracil-containing upstream primer and an unlabeled downstream primer. Fragments were treated with *E. coli* UDG, leaving an intact AP site. Because the AP site is relatively unstable at high temperature, part of the denatured strand was broken into a 5′-6-FAM-23 nt fragment and an unlabeled 517 nt fragment, the latter of which could be detected on the gel (Figure 4, lane 3). The rest of the reconstitution process was performed by adding potent BER repair homologs from *D. radiodurans*. drXth, an AP endonuclease from *D. radiodurans* (11), incises the 3′-end of the AP site, created a 5′-6-FAM-23 nt fragment containing a 3′-OH terminus and an unlabeled 517 nt fragment containing a 5′-dRP group. *D. radiodurans* DNA polymerase I (drPolA) then mediates DNA synthesis from the 3′-OH group and promotes the displacement of the downstream DNA strand, creating a 5′ flap with a 5′-dRP end. When the free end is long enough (our biochemical assay indicated that a free 5′ tail of at least 3−4 nt is required for digestion initiation; Supplementary Figures S7 and S8), drRecJ may recognise the 5′-dRP group, and digest the 5′ flap efficiently into DNA duplex regions of a few hundred nucleotides, leaving a nick containing 5′-P. This nick is ligated by drLigA, the major DNA ligase in *D. radiodurans* (41). In the present study, because full-length WT drPolA exhibits strong 5′−3′ exonuclease activity, which will digest primer DNA from the 5′ end and mask its synthetic activity, the completely repaired strand (5′-6-FAM-labeled 541 nt fragment) is difficulty to monitor (Figure 4, lanes 14 and 15). Therefore, the drPolA(D119/120A) variant lacking 5′−3′ exonuclease activity or the drPolA-C variant in which the 5′−3′ exonuclease domain is truncated was used instead for the 3′-OH termini extension. Completely repaired strands could be detected only in the presence of drPolA(D119/120A), drRecJ and drLigA (Figure 4, lane 11), indicating that drRecJ contributes to the repaired product formation. It is worth noting that the synthesis activity of drPolA-C was slightly weaker than that of the full-length drPolA(D119/120A). Since the formation of the 5′ flap strand is mediated by displacement synthesis by PolA, weaker synthesis activity might have a negative impact on 5′ flap formation or repaired product ligation, and this could explain why we failed to obtain fully repaired product using drPolA-C (Figure 4, lane 13).

**Figure 5.**
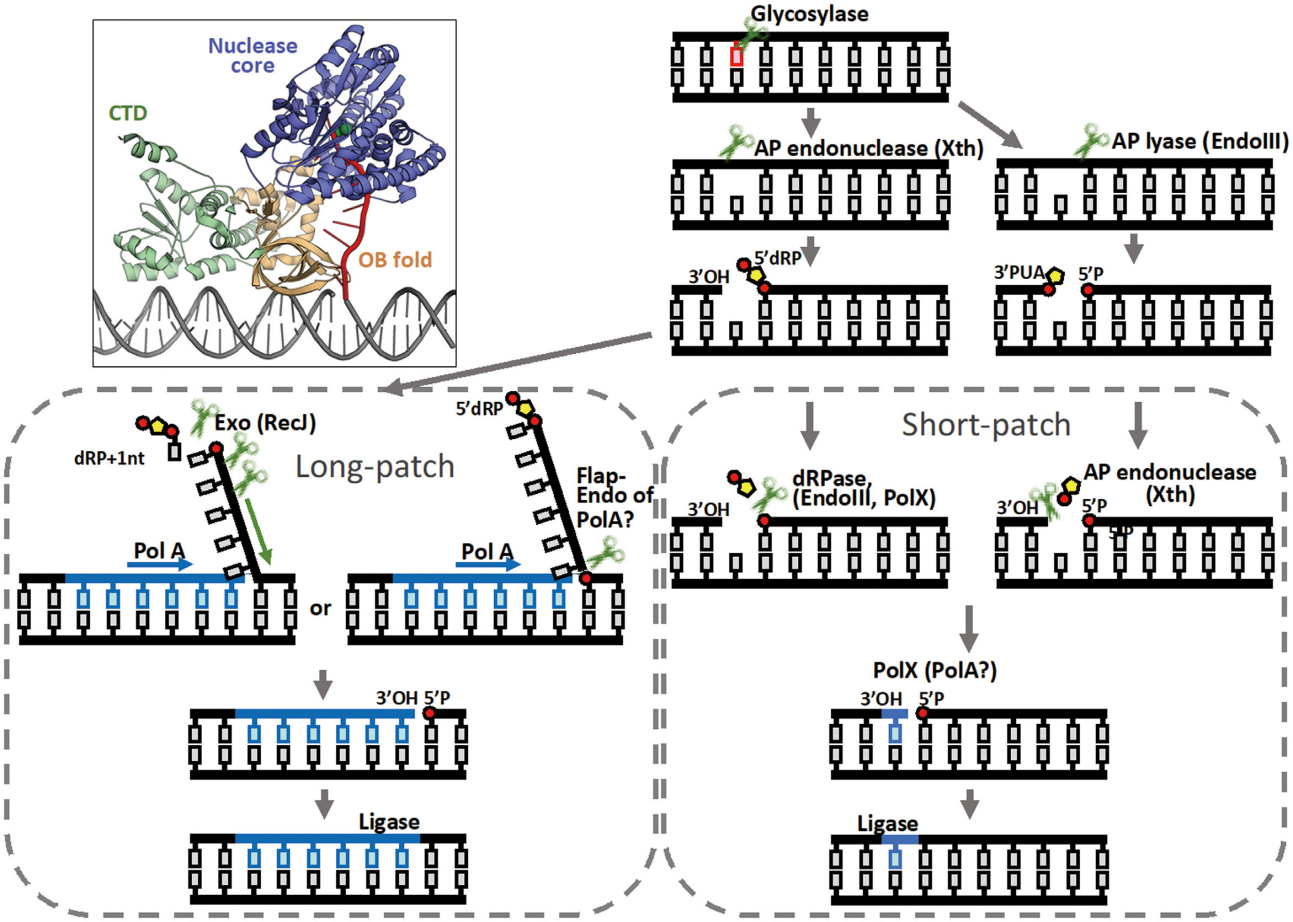
Schematic of the BER pathways of *D. radiodurans*. Damaged bases are first removed by glycosylases. AP endonuclease Xth, or AP lyse endonuclease III, induces a nick in the bond at the 5′-side of the abasic site and creates termini with 3′-OH and 5′-dRP, or at the 3′-side of the abasic site and creates termini with 3′-PUA and 5′-P. 5′-dRP can be removed directly by dRPase, such as endonuclease III and PolX from the 3′-side of the abasic site, and 3′-PUA can be removed directly by AP endonuclease Xth from the 5′-side of the abasic site. The repair synthesis involves the insertion of one nucleotide by a gap-filling polymerase, such as PolX, or PolA, and the ligation by DNA ligase. In the long-patch BER pathway, a processive polymerase, such as PolA, mediates DNA synthesis and promotes a displacement of the downstream DNA strand. The displaced flap structure is digested by 5′-3′ exonuclease RecJ or a flap-endonuclease (such as PolA), with the resulting nick ligated by DNA ligase. A structure model of RecJ participating in long-patch BER was provided based on real crystal structures (PDB code: 5F55). Only the DNA duplex (coloured as grey) is artificially added here.

Based on previous data and our structural/biochemical results, we produced a schematic representation of the *D. radiodurans* BER pathways (Figure 5). Damaged bases are first removed by an ensemble of glycosylases (7). AP endonuclease Xth (DR0354) (10,11) or AP lyse endonuclease III (DR0928, DR2438 or DR0289) (8) induces a nick in the bond at the 5′-end of the AP site and creates termini with 3′-OH and 5′-dRP, or at the 3′-end of the AP site it creates termini with 3′-PUA and 5′-P. Removal of a dRP moiety is the main difference between the short- and long-patch BER pathways. When 5′-dRP or 3′-PUA can be removed directly by a dRPase, such as endonuclease III (8) and PolX (DR0467) (21) from the 3′-end of the AP site, or AP endonuclease Xth (DR0354) from the 5′-end, the repair synthesis involves the insertion of one nucleotide by a gap-filling polymerase such as PolX, and ligation by DNA ligase (DR2069). PolA may also partake in the gap-filling process, but PolA (DR1707) from *D. radiodurans* is a highly processive polymerase that prefers to synthesise long strands rather than a single nucleotide (11), and is therefore not likely to contribution here. Thus, when immediate ligation is impossible, the long-patch BER pathway would take over the remaining process. PolA (DR1707) mediates DNA synthesis and promotes downstream DNA strand displacement. The displaced flap structure is digested by the 5′−3′ exonuclease activity of RecJ (DR1126), with the resulting nick ligated by DNA ligase. The N-terminal domain of drPolA, a homolog of which was identified as a flap endonuclease in *E. coli*, may also participate in this process.

## DISCUSSION

A few decades ago, Dianov and Lindahl adopted a short dsDNA substrate containing a dU inserted within one strand to reconstitute the *E. coli* BER pathway *in vitro* (42). *E. coli* uracil-DNA glycosylase, *E. coli* polymerase I (PolA), *E. coli* AP endonuclease IV, *E. coli* RecJ and *E. coli* DNA Ligase were used for the reconstitution assay. The size of repair patches was estimated based on the overall incorporation of radioactive nucleotides. The authors noticed filling in with lots of single nucleotides (minimal patch size) when the ecRecJ enzyme was added. Therefore, they concluded that ecRecJ is involved in the removal of the 5′dRP group as part of the short-patch BER pathway. However, more detailed analysis of the DNA product of ecRecJ is lacking. A few years later, researchers from another group failed to detect dRPase activity of ecRecJ on pre-incised AP DNA substrates (a short dsDNA substrate containing a dU inserted within one strand treated sequentially with UDG and human APE, creating a terminus with 3′OH and 5′-dRP) (43). Based on their results, the authors concluded that ecRecJ cannot be a major player in the short-patch BER pathway. A subsequent study on the *Chlamydiophila pneumoniae* BER pathway revealed that cpRecJ also had little effect on the short-patch BER (44).

Here, genetic assays confirmed that RecJ is important for the *D. radiodurans* BER pathway. However, drRecJ is very unlikely to act in the short-patch BER pathway for three major reasons:

1. In the short-patch BER pathway, a canonical 5′-dRP lyase removes the 5′-dRP group, a polymerase fills the gap with a single nucleotide, and a DNA ligase seals the nick. Although drRecJ preferentially binds and digests 5′-dRP-modified ssDNA substrates, both our structural and biochemical data indicate that drRecJ is unlike canonical 5′-dRP lyases that break the second phosphodiester bond, leaving a 5′-dRP linked to a nucleotide; rather, drRecJ breaks the first phosphodiester bond and leaves only a dRP group. The drRecJ catalytic pattern can be explained by the structural features around its active sites. The 5′-P binding pocket and the extra space suitable for 5′-dRP occupancy located near that pocket plain the preferential binding of 5′-dRP substrates. Additionally, stable π-stacking between the first base and the tyrosine in the catalytic core facilitates in-line attack of the second phosphodiester bond instead of the first phosphodiester bond on 5′-dRP substrates.
2. RecJ requires a ssDNA with a free 5′ end to initiate digestion. PolA or other helicases can assist initiation by creating a 5′ flap or 5′ overhang structure, which is a key step in the long-patch BER pathway. Based on the actual RecJ-DNA complex structures from this and previous studies, we generated a 3D model of RecJ participating in the long-patch BER pathway (Figure 5). The architecture of both the nuclease core and the oligonucleotide/oligosaccharide-binding (OB) fold allow only ssDNA to be tightly bound and fed into the catalytic site. Furthermore, the catalytic site is deeply embedded in the nuclease core at least four free bases away from the enzyme surface. Ten free bases are preferred for tightly binding the OB fold. Our biochemical results indicate that a free 5′ end with at least four free bases (or three nt plus a 5′dRP group) is required for efficient digestion initiation (Supplementary Figures S7 and S8). The requirement for a free 5′ end for efficient digestion was also reported for RecJs from other bacteria, such as *Haemophilus influenza* RecJ and *C. pneumoniae* RecJ (44,46). Additionally, the ecRecJ enzyme requires single-stranded tails (seven nt or longer) for robust binding and digestion (45). Our biochemical results also indicate that without the help of drPolA, drRecJ exonuclease activity on pre-incised AP DNA substrates was blocked (Supplementary Figure S8). Therefore, a 5′dRP group embedded within a DNA duplex is clearly not efficiently captured into the drRecJ catalytic site, unless other enzymes help to create a 5′ flap structure, a process that only occurs in long-patch BER pathway.
3. RecJ is a highly processive ssDNA exonuclease, and once initiated, it can cut DNA into duplex regions from substrates hundreds of nucleotides in length. Such a feature was also addressed in a study on *E. coli* RecJ (47). It seems that despite lacking a canonical helicase domain, RecJ can locally melt dsDNA and bind to the resulting 5′-ssDNA for resection (34) (Supplementary Figures S7 and S8). For the BER reconstitution assay, we initially selected a short substrate (32 bp) similar in size to that used by Dianov and Lindahl in their ecRecJ BER reconstitution assay, and labeled both ends of the AP site-containing strand. Unlike their results, we found that with the help of drPolA, drRecJ could completely digest the 5′dRP fragment before DNA ligase acts (products on the gel were all single nucleotides labeled at the 3′ tail; data not shown). We subsequently designed a new system using a longer substrate (541 bp) and finally reconstituted the BER pathway *in vitro*. Therefore, it is hard to explain how such a processive exonuclease can be involved in the short-patch BER pathway, which is believed to cease digestion after cutting only one dRP group.

Collectively, since drRecJ does not have the capability to capture the dRP group within dsDNA, and does not have a preference for removing a single dRP group, we believe that it is likely to participate in the long-patch BER pathway rather than the short-patch BER pathway in *D. radiodurans*.

However, there has been much controversy over the years regarding the role of RecJ in the BER pathway. Thus, we cannot rule out the possibility that different RecJs behave differently during BER. Sequence alignment revealed that the ssDNA binding channel, 5′-P binding pocket and 5′-dRP sensor motifs within the RecJ nuclease core are highly conserved, indicating that RecJs might share similar 5′-dRP substrate catalytic and translocation mechanisms. However, detailed analysis by MS should be conducted to further confirm that they are not canonical 5′-dRP lyases. Although biochemical evidence based on numerous bacterial RecJs indicates that they are ssDNA-specific exonucleases, it is also possible that RecJs may be able to cut the dRP group within a DNA duplex and act in the short-patch BER pathway through other novel strategies, potentially via their non-conserved C-terminal domain, or with the help of other unknown protein partners.

## DATA AVAILABILITY

The coordinates and structure factors have been deposited to Protein Data Bank with accession codes 6LRD.

## Supplementary Material

gkaa714_Supplemental_File
