# Supplementary material for: Participation of RecJ in the base excision repair pathway of Deinococcus radiodurans
Source: Nucleic Acids Res. 2020 Sep 1;48(17):9859–71. doi: 10.1093/nar/gkaa714 (PMC7515722; doi:10.1093/nar/gkaa714)
Supplement: gkaa714_Supplemental_File [file gkaa714_supplemental_file.pdf]

Supplemental materials

Participation of RecJ in the base excision repair pathway of *Deinococcus radiodurans*

Kaiying Cheng[#], Ying Xu[#], Xuanyi Chen, Huizhi Lu, Yuan He, Liangyan Wang and Yuejin Hua*

MOE Key Laboratory of Biosystems Homeostasis & Protection, Institute of Biophysics, College of Life Sciences, Zhejiang University, China

[#] These two authors contribute equally.

* To whom correspondence should be addressed. Tel: 86-571-86971703; Fax: 86-571-86971703; Email: yjhua@zju.edu.cn

Table S1 Primers or oligonucleotides used in this study.

Oligonucleotide name	Sequence (5'→3') *
**polX_F(NdeI)**	TTTTTTCATATGACCCTGCCGCCCG
**polX_R(BamHI)**	TTTGGATCCTTATGCACGGTCCGCC
**polA_F(NdeI)**	TTTTTTCATATGGCCGACGCTTCCCCAG
**polA_R(BamHI)**	TTTGGATCCTCACTTCGTGTCAAACCAG
**polA-C_F(NdeI)**	TTTTTTCATATGGTCTGGGGCTACGTCCT
**polA (D119/120A)F**	GAGGCGATCACGGCGGCGGCCTCGTAGC
**polA (D119/120A)R**	GCTACGAGGCCGCCGCCGTGATCGCCTC
**LigA_F(NdeI)**	GGAATTCCATATGATGACCGACGCGCCG
**LigA_ R(BamHI)**	CGGGATCCTCAGCTTTCAGCGGGGG
**Xth_F (NdeI)**	GGAATTCCATATGTTGAGCCTCCTTGCCCCA
**Xth_R (BamHI)**	CGGGATCCTCATTCAGATTCCAGCTCCACC
**KY04 (3'FAM)**	TTTTTTTTTTTTTTAGTACTCGAGTGCTGGCGAGCACTCGAGTACT
**KY05 (3'FAM)**	AGTACTCGAGTGCTGGCGAGCACTCGAGTACT
**KY08 (3'FAM)**	AAAAAAAAAAAAAAAAAAAA
**KY10 (3'FAM)**	5'P-AAAAAAAAAAAAAAAAAAAA
**KY11 (3'FAM)**	5'P-dSpacer-AAAAAAAAAAAAAAAAAAAA
**KY18 (3'FAM)**	TTTTTTTAGTACTCGAGTGCTGGCGAGCACTCGAGTACT
**KY19 (3'FAM)**	TTTTTAGTACTCGAGTGCTGGCGAGCACTCGAGTACT
**KY20 (3'FAM)**	TTTTAGTACTCGAGTGCTGGCGAGCACTCGAGTACT
**KY21 (3'FAM)**	TTTAGTACTCGAGTGCTGGCGAGCACTCGAGTACT
**KY22 (3'FAM)**	TTAGTACTCGAGTGCTGGCGAGCACTCGAGTACT
**KY23**	AAAAAAAAAAAAAAAAAAAA
**KY24**	5'dSpacer-AAAAAAAAAAAAAAAAAAAA
**KY26**	5'P-dSpacer-AAAAAAAAAAAAAAAAAAAA
**KY27 (5'FAM)**	AACTTTAAGAAGGAGATATACCAUGGGCAGCAGCCATCAT
**KY27_PCR_R**	TCGAGTGCGGCCGCAAGCTT
**KY25**	5'P-dSpacer-TTTTT
**KY28 (3'FAM)**	5'P-dSpacer-CTGAGCGAGAGTCTTGC
**KY29**	GCAAGACTCTCGCTCAGTTGATTACCACCATTTTTGGTGGTAATCA
**KY30**	GCAAGACTCTCGCTCAGTTGATTACCACCATTTTTGGTGGTAATCAA
**KY31**	GCAAGACTCTCGCTCAGTTGATTACCACCATTTTTGGTGGTAATCAAC
**KY32**	GCAAGACTCTCGCTCAGTTGATTACCACCATTTTTGGTGGTAATCAACT
**KY33**	GCAAGACTCTCGCTCAGTTGATTACCACCATTTTTGGTGGTAATCAACTG

Table S2 Kinetic parameters of drRecJ protein on different substrates.

Substrate	K_m (nM)	k_{cat} (min^{-1})	k_{cat}/K_m (μM^{-1} min^{-1})
**KY08 ((dA)$_{20}$)**	100.3 ± 5.5	1.15 ± 0.02	11.5±0.9
**KY10 (P(dA)$_{20}$)**	97± 6.3	1.18± 0.03	12.2±1.1
**KY11 (PdSpacer(dA)$_{20}$)**	81± 9.1	1.21± 0.05	15.1±2.1

10 nM drRecJ was incubated with saturated substrate (KY08, KY10 and KY11 0-2 μM) in digestion buffer at 30°C for 20 min, products were resolved on 15% denature gel. All reactions were independently repeated at least three times. The k_{cat} and K_m were derived from generalized nonlinear least squares using the Michaelis-Menten equation, from which the apparent second order rate constant (k_{cat}/K_m) was determined from a plot of the normalized initial rate (v/[E]) versus the substrate concentration ([S]).

Figure S1 The H_2O_2 survival rate.

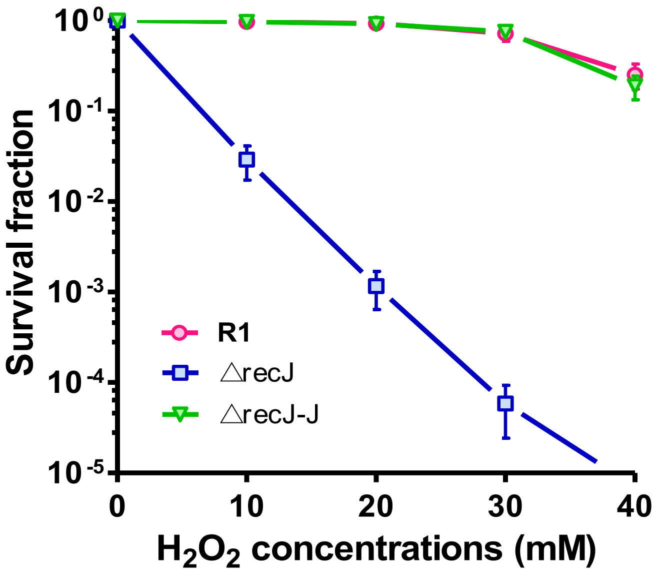

Wild type strain (R1), drRecJ deletion mutant *△recJ* and *recJ* complemented strain *△recJ-J*, were treated with different concentrations of H_2O_2. Cells were diluted and plated on plates. Colonies were counted after 3 days. The survival fraction curves were plotted using GraphPad Prism 6 software.

Figure S2 The raw data of ARP-DNA Standard and experimental groups.

The OD_{450} values of ARP-DNA Standard and Experimental groups

ARP-DNA Standard (X AP Sites per 100,000 bp DNA)							
Group	2	4	8	16	24	32	40
1	0.010232	0.084725	0.214515	0.486342	0.913765	1.222456	1.644667
2	0.012974	0.039003	0.179554	0.57256	0.877424	1.325239	1.669034
3	0.030235	0.038923	0.244624	0.627146	0.987084	1.395764	1.740234
Experimental groups							
Group	WT	WT H_2O_2 treated	ΔrecJ	ΔrecJ H_2O_2 treated	ΔrecJ-J	ΔrecJ-J H_2O_2 treated	Control (no (AP site)
1	0.176098	0.584517	0.720395	2.142829	0.185997	0.61482	0
2	0.271928	0.659889	0.861646	2.073565	0.277303	0.532305	0
3	0.313664	0.438265	0.85729	1.939541	0.241589	0.381523	0

OD values were measured at 450 nm. The no AP site DNA was set as control. The final control values were reset to zero after measurements and all the other values were recalculated based on the control values. All DNA samples and standards were assayed using three duplicates.

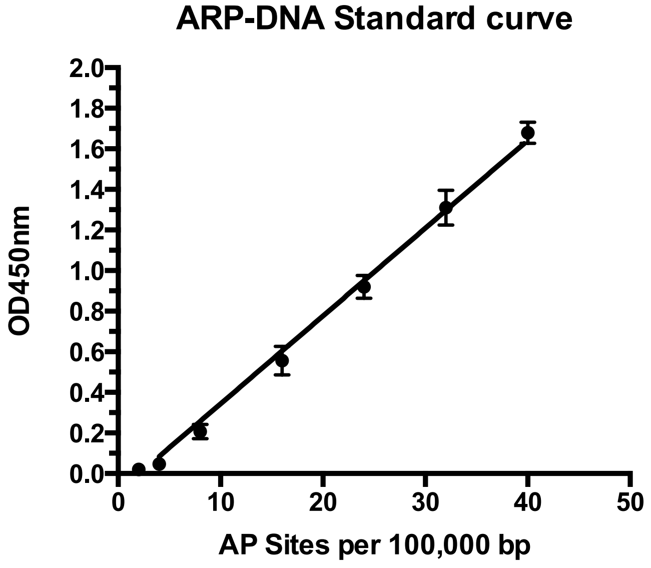

1/slope=23.10.

AP site numbers per 100,000 bp of experimental groups

	Without treatment			Treated with H_2O_2		
Group	1	2	3	1	2	3
WT	4.067864	6.281537	7.245638	13.50234	15.24344	10.123920
ΔrecJ	16.641120	19.904020	19.803400	49.49935	47.89935	44.803400
ΔrecJ-J	4.296531	6.405699	5.580706	14.20234	12.29625	8.813181

The AP site numbers per 100,000 bp of experimental groups were calculated according to the ARP-DNA Standard curve shown above.

Figure S3 Mass spectrum analysis of KY26 digested by drRecJ.

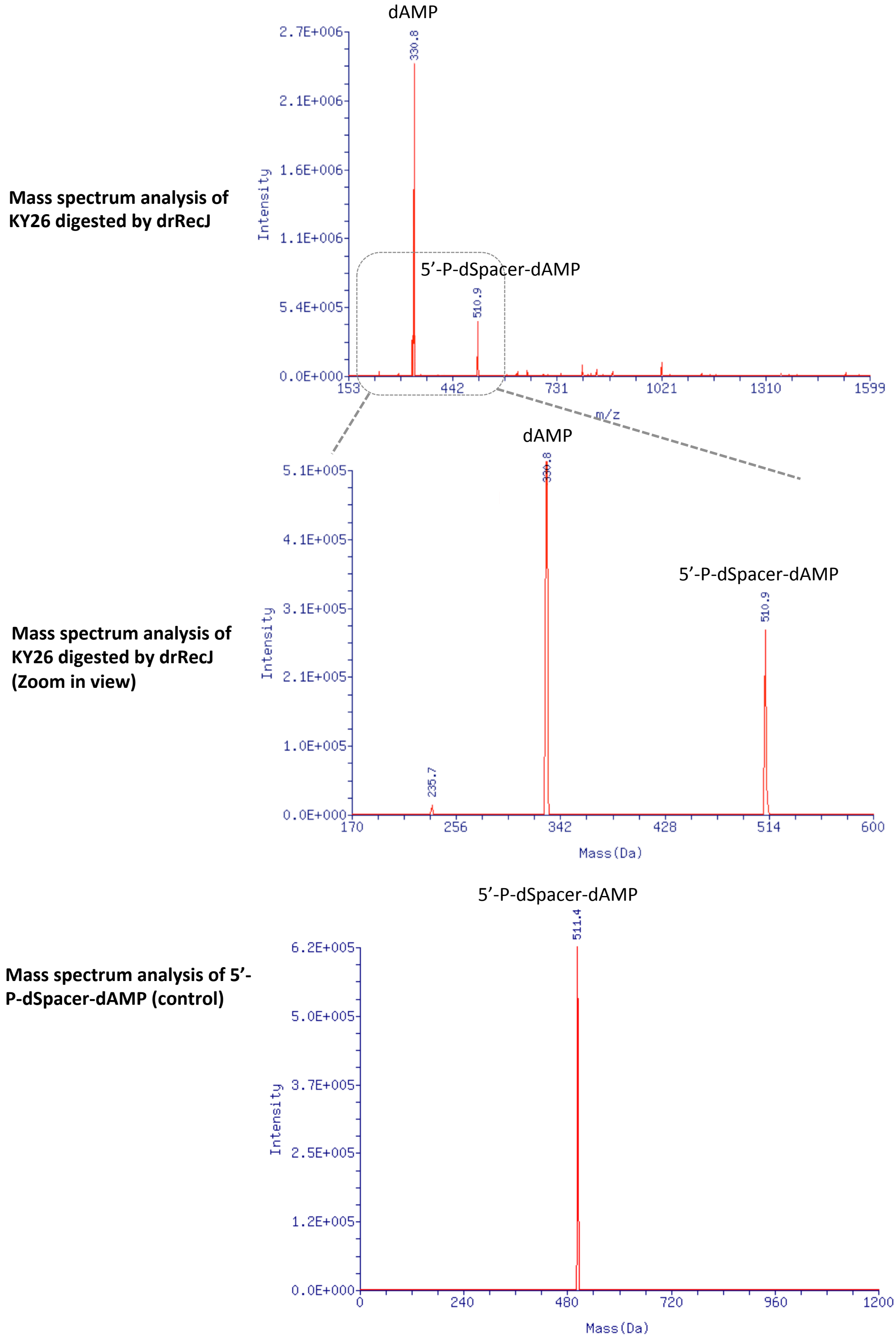

The mass spectrum analysis of KY26 digested by drRecJ identified two obvious signals, which are dAMP (~330 Da) and 5'-P-dSpacer-dAMP (~510 Da), respectively. There is no apparent signal for 5'-P-dSpacer group, which molecular weight supposed to be 180 Da.

Figure S4 DNA binding capability of drRecJ mutant proteins.

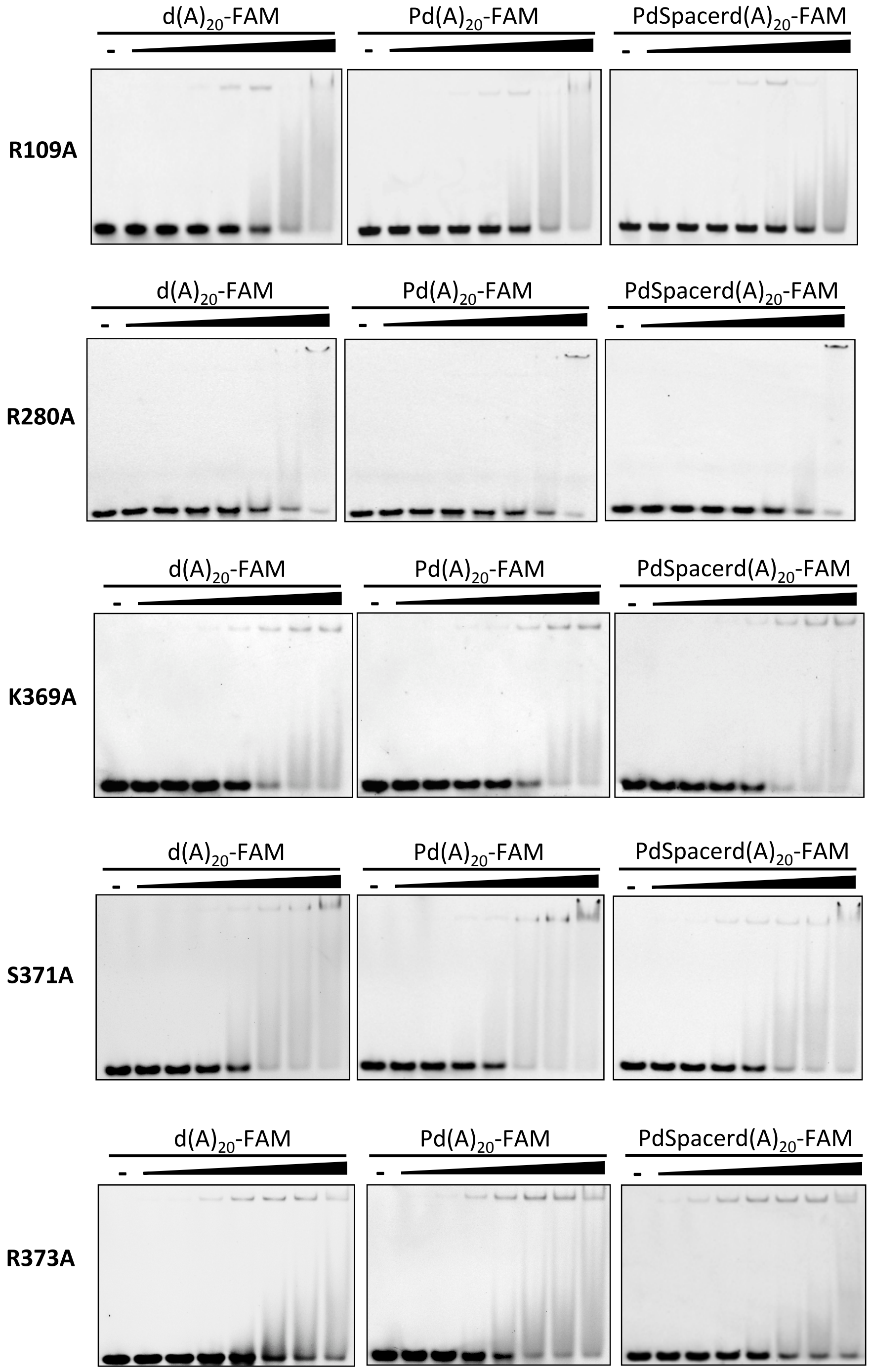

Different DNA substrates with various 5' modifications (10 nM), as shown at the top of the panel, were incubated with gradient diluted drRecJ mutants. Among them, the concentrations of R109A, K369A and S371A are 0, 2.5, 5, 10, 20, 40, 80 and 160 nM, while the concentrations of R280A and R373A are 0, 5, 10, 20, 40, 80, 160 and 320 nM. The formed complexes were separated by native-TBE gel.

Figure S5 Nuclease activity of drRecJ mutant proteins.

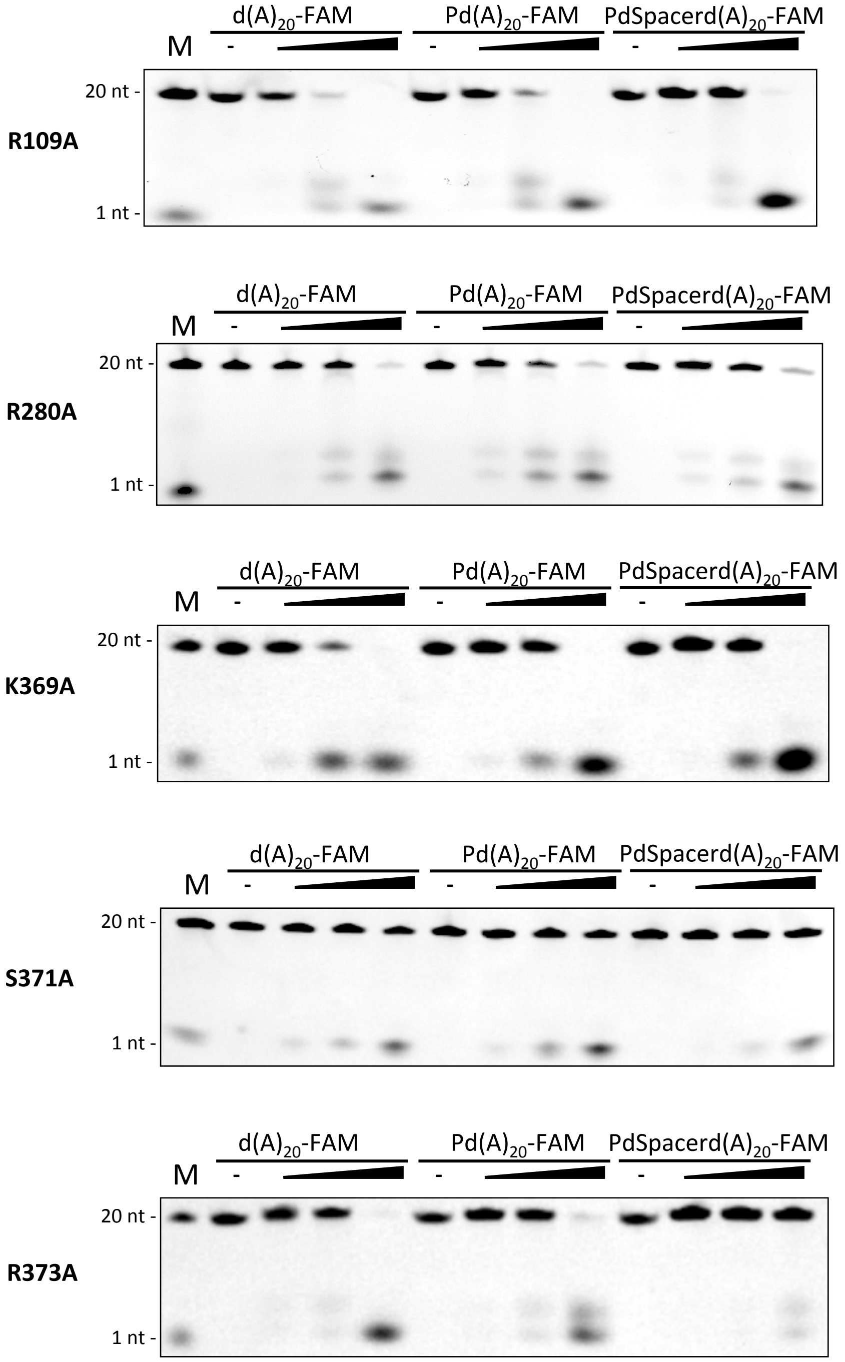

Different DNA substrates with various 5' modifications (100 nM), as shown at the top of the panel, were incubated with gradient diluted drRecJ mutants in the presence of 100 nM Mn^{2+}, and then separated by denatured PAGE gel. Among them, the concentrations of R280A, S371A and R373A are 0, 10, 20, and 40 nM. The concentrations of K369A are 0, 20, 40, and 80 nM. And the concentrations of R109A are 0, 40, 80, and 160 nM.

Figure S6 The quality control data (mass spectrum results) of main oligos in this study.

KY08: AAAAAAAAAAAAAAAAAAAAA-FAM

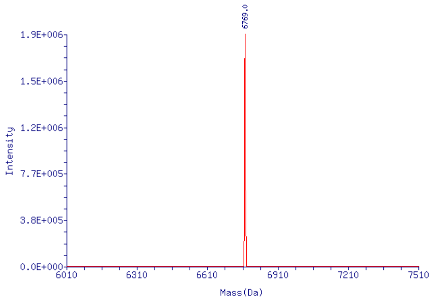

KY10: 5’P-AAAAAAAAAAAAAAAAAAAAA-FAM

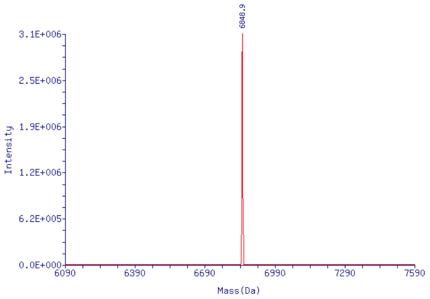

KY11: 5’PdSpacer-AAAAAAAAAAAAAAAAAAAAA-FAM

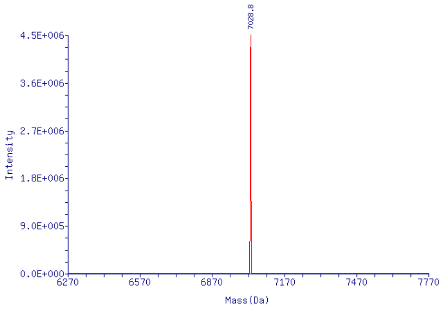

KY23: AAAAAAAAAAAAAAAAAAAAA

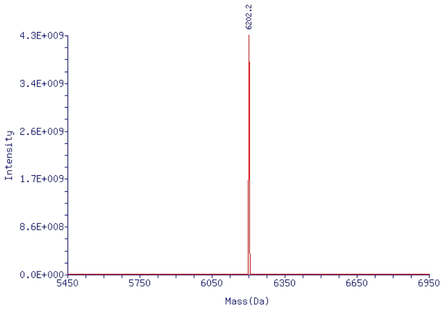

KY24: dSpacer-AAAAAAAAAAAAAAAAAAAAA

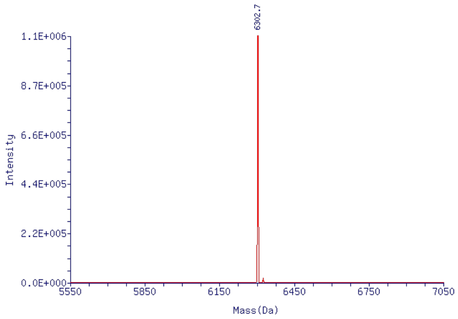

KY25: 5’P-dSpacer-TTTTTT

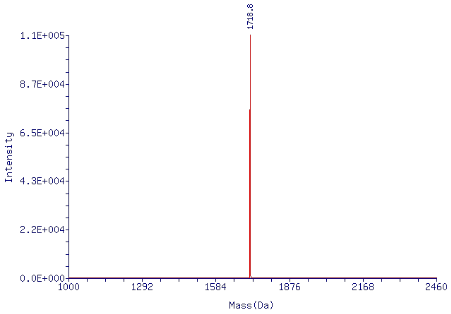

KY26: 5’P-dSpacer-AAAAAAAAAAAAAAAAAAAAA

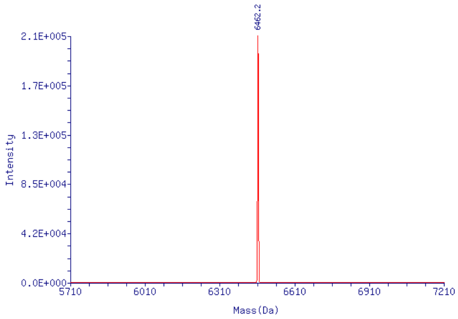

KY28: 5’P-dSpacer-CTGAGCGAGAGTCTTGC-FAM

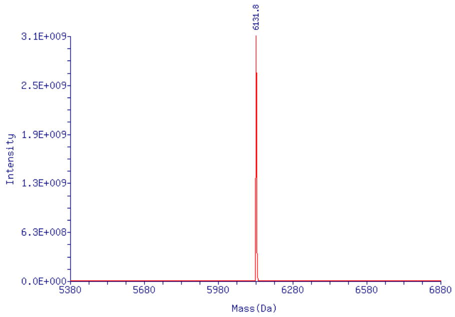

Figure S7 The length of 5’ free end required for efficient digestion by drRecJ

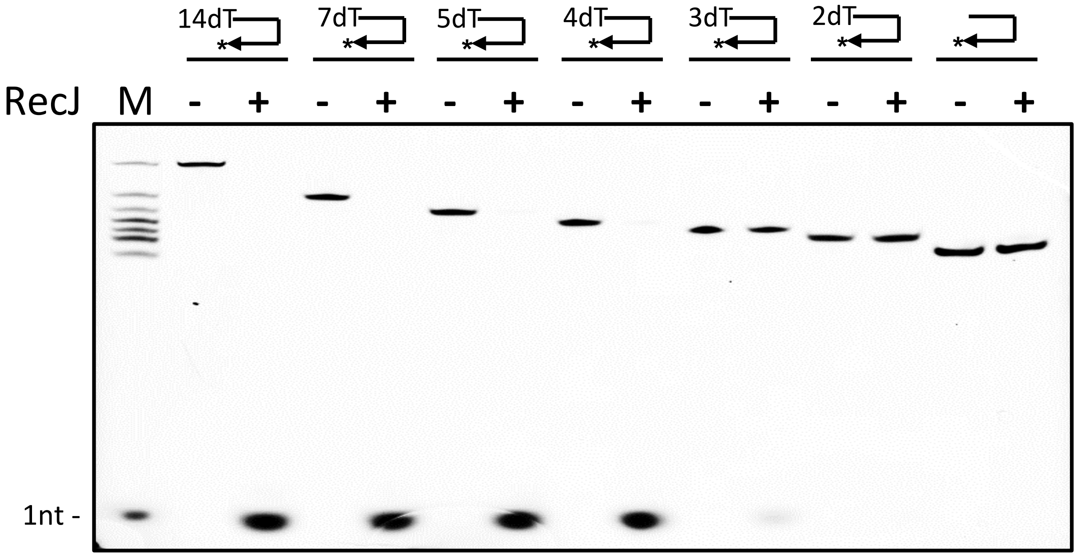

100 nM dsDNA with different 5’ ssDNA overhang lengths were incubated by drRecJ at 30°C for 20 min and their digestion efficiencies were compared. Reaction products were resolved on denatured PAGE gel. M, marker (was created by a mixture of different lengths of substrates and 1nt product).

Figure S8 The length of 5’ -dRP free end required for efficient digestion by drRecJ

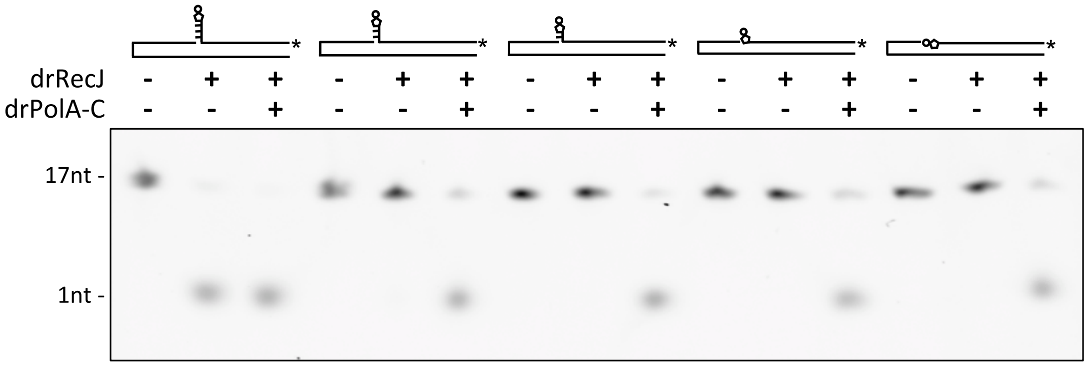

100 nM substrates with different 5’ flap lengths (3 nt + 5’-P-dSpacer, 2 nt + 5’-P-dSpacer, 1 nt + 5’-P-dSpacer, 5’-P-dSpacer group or no flap structure) were incubated by drRecJ at 30°C for 20 min, in the absence or presence of drPolA nuclease truncated mutant drPolA-C. Reaction products were resolved on denatured PAGE gel.
